# Characteristics of Plasmids in Multi-Drug-Resistant *Enterobacteriaceae* Isolated during Prospective Surveillance of a Newly Opened Hospital in Iraq

**DOI:** 10.1371/journal.pone.0040360

**Published:** 2012-07-11

**Authors:** Xiao-Zhe Huang, Jonathan G. Frye, Mohamad A. Chahine, LaShanda M. Glenn, Julie A. Ake, Wanwen Su, Mikeljon P. Nikolich, Emil P. Lesho

**Affiliations:** 1 Bacterial Diseases Branch, Walter Reed Army Institute of Research, Silver Spring, Maryland, United States of America; 2 Bacterial Epidemiology and Anti-drug Resistant Unit, Agriculture Research Service, U.S. Department of Agriculture, Athens, Georgia, United States of America; 3 Infectious Diseases Service, Walter Reed Army Medical Center, Washington, D.C., United States of America; University of Maryland, United States of America

## Abstract

**Background:**

Gram-negative multidrug-resistant (MDR) bacteria are major causes of nosocomial infections, and antibiotic resistance in these organisms is often plasmid mediated. Data are scarce pertaining to molecular mechanisms of antibiotic resistance in resource constrained areas such as Iraq.

**Methodology/Principal Findings:**

In this study, all MDR *Enterobacteriaceae* (n = 38) and randomly selected non-MDR counterparts (n = 41) isolated from patients, healthcare workers and environmental surfaces in a newly opened hospital in Iraq were investigated to characterize plasmids found in these isolates and determine their contribution to antibiotic resistance. Our results demonstrated that MDR *E. coli* and *K. pneumoniae* isolates harbored significantly more (≥3) plasmids compared to their non-MDR counterparts, which carried ≤2 plasmids (p<0.01). Various large plasmids (∼52 to 100 kb) from representative isolates were confirmed to contain multiple resistance genes by DNA microarray analysis. Aminoglycoside (*acc, aadA, aph, strA/B, and ksgA*), β-lactam (*bla*_TEM1_, *bla*_AMPC_, *bla*_CTX-M-15_, *bla*_OXA-1_, *bla*_VIM-2_ and *bla*_SHV_), sulfamethoxazole/trimethoprim (*sul*/*dfr*), tetracycline (*tet*) and chloramphenicol (*cat*) resistance genes were detected on these plasmids. Additionally, multiple plasmids carrying multiple antibiotic resistance genes were found in the same host strain. Genetic transfer-associated genes were identified on the plasmids from both MDR and non-MDR isolates. Seven plasmid replicon types (FII, FIA, FIB, B/O, K, I1 and N) were detected in the isolates, while globally disseminated IncA/C and IncHI1 plasmids were not detected in these isolates.

**Conclusions/Significance:**

This is the first report of the characteristics of the plasmids found in *Enterobacteriaceae* isolated following the opening of a new hospital in Iraq. The information provided here furthers our understanding of the mechanisms of drug resistance in this specific region and their evolutionary relationship with other parts of world. The large plasmids, carrying resistance genes and transfer-associated genes, may be potential factors for regional dissemination of antibiotic resistance.

## Introduction

Antibiotic resistance in bacterial pathogens is steadily increasing and recognized as one of the greatest threats to global public health [1]. However, the genetic features of the drug resistance in Gram-negative *Enterobacteriaceae* from certain geographic locations such as Iraq have not been defined due to constrained resources.

There are several factors responsible for dissemination of antimicrobial resistance genes among bacterial strains, and plasmid-mediated transfer has been considered one of the most important mechanisms for the horizontal transfer of multidrug resistance [2], [3], [4]. In Gram-negative bacteria, genes such as *bla*_SHV_, *bla*_TEM_, *bla*_CTX_ and *bla*_AMPC_ presented in *E. coli*, *K. pneumoniae* and *Acinetobacter spp*. encode extended-spectrum β-lactamases (ESBLs) that are often located on plasmids [5]. The genes found in many Gram-negative bacilli including *Enterobacteriaceae*, *Pseudomonas aeruginosa* and *Acinetobacter spp.* encode class A, B and D β-lactamases that mediate resistance to various β-lactam antibiotics have also been found on plasmids [6], [7], [8]. Additionally, plasmids conferring resistance to quinolones and/or aminoglycosides have been reported [5], [9].

Recently, a DNA microarray assay for 775 antimicrobial resistance related genes was developed [10]. This method has been successfully used to detect antimicrobial resistance genes in a variety of Gram-positive and -negative bacteria [11]. PCR-based replicon typing (PBRT) has also been used to categorize plasmids found in *Enterobacteriaceae* strains which have MDR genes located on plasmids [12], [13]. A recent review summarized the major plasmid families that are currently emerging in MDR *Enterobacteriaceae* strains isolated in several parts of the world (with the exception of the Middle East) including those conferring resistance to important antibiotics such as extended-spectrum cephalosporins, fluoroquinolones and aminoglycosides [5]. Certain replicon types were found to be associated with MDR as well as with bacterial disease outbreaks [8], [13]. The ability to identify and categorize plasmids on the basis of their phylogenetic relatedness enables the analysis of their distribution in nature and their relationship to bacterial hosts, and provides insight into their evolutionary origins. In turn, this can be useful for epidemiologic surveillance and the development of strategies to prevent their spread [5], [14].

In this study, we investigated the presence of plasmids in antibiotic resistant isolates collected from a new hospital in Iraq. The isolates were taken during the surveillance of patients, healthcare workers and environmental surfaces for Gram-negative MDR bacteria before and after the opening of this new hospital in eastern Iraq from October 2007 to May 2008 [15], [16]. The sampling scheme employed allowed us to explore the effects of early molecular events such as the presence or absence of plasmids and antibacterial resistance in the *Enterobacteriaceae* present during colonization of a new hospital. Plasmid profile analysis, gene analysis by microarray and plasmid replicon typing were conducted to identify correlations between plasmids and drug resistance, to evaluate the potential horizontal transfer of plasmids among these organisms and to understand the evolutionary background of the plasmids from this specific geographic region by comparing them with drug resistant plasmids found in other parts of the world.

## Materials and Methods

### Ethics Statement

This study was approved by the Internal Review Board at Walter Reed Army Institute of Research (WRAIR), under protocol number1496 for using bacterial isolates from human patients.

### Setting and Patients

Details of the hospital setting and patients were described by Lesho, EP *et al.* elsewhere [15]. Briefly, the facility was located in eastern Iraq and had a staff of 55 health care workers, two fully equipped operating suites, a dual recovery-intensive care room with two permanent beds and a ward with four permanent medical-surgical beds. It also had a two-bed trauma bay, four ambulatory examination rooms, and a basic laboratory with a 10–20-unit blood supply, an enhanced pharmacy and digital x-ray capability. Major groups of patients in this hospital included Iraqi military and Iraqi civilians, U.S. military and U.S. civilians, and coalition military forces from El Salvador, the Republic of Georgia, Kazakhstan and Poland. Multidrug resistant organism (MDRO) surveillance was undertaken as a quality improvement and infection control effort.

### Prospective Surveillance of MDRO for Infection Control in the Hospital: Bacterial Collection

**Figure 1:**
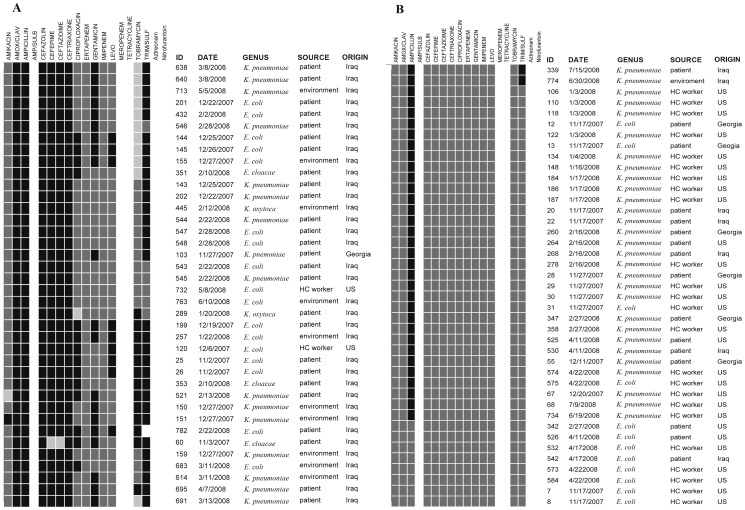
Antibiograms and basic epidemiological characteristics of both MDR (A) and non-MDR (B) bacteria isolates. Solid black: resistance to the antibiotics; Grey: susceptible to the antibiotics; Light grey: intermediate; White: N/A; ID: strain identification. HC worker: Healthcare worker.

Details of the surveillance methods have been published elsewhere [16]. Briefly, patients, personnel and environmental surfaces were prospectively and routinely sampled before the opening of the new facility and for the following six months. The opening date of the hospital was on December 7, 2007. Sampling was performed with swab transport systems containing liquid Amies medium without charcoal (Copan Venturi Transystem; Becton Dickinson MaxV[+]). Patients admitted and/or treated in the operating room or trauma bay were sampled within 24 hours, and on discharge as well. Outpatients with skin infections were also sampled. Swab samples were taken of the axilla and groin to detect skin colonization. Swab samples were also taken of all open wounds, regardless of clinical evidence of infection. All hospital personnel (medical, surgical and administrative) were sampled in the axilla and groin to detect skin colonization. Existing hopital personnel were sampled at the time of the new facility opening and every 2–4 weeks thereafter. New personnel were sampled on arrival at the facility and every 2–4 weeks thereafter. To collect environmental samples, swabs were aseptically removed from the sterile container and premoistened by dipping in sterile nutrient-buffer solution. Using S strokes and firm, consistent pressure, the surfaces were wiped using enough vertical and horizontal strokes to cover a representative majority of the sample area. Swabs were rolled so that all sides and areas of the swab tip were used. Environmental sampling was perfomed in 22 patient flow areas. Every MDR *Enterobacteriaceae* that was isolated during surveillance (n = 38) and 41 randomly selected non-MDR *Enterobacteriaceae* counterparts were collected for detailed analyses of their plasmid genetic features. Figure 1 provides basic epidemiological information of the bacterial isolates studied.

### Antibiotic Susceptibility Tests (CLIA certified) [17]

Antibiotic Susceptibility Tests were performed on a Phoenix NMIC/ID133 panel (Becton Dickinson and Company, Franklin Lakes, NJ). Susceptibility was determined according to CLSI M-100-S-19, Vol 29, No. 3 2009 [17]. Bacteria were considered MDR if they were resistant to three or more classes of antibiotic agents. Isolates that were resistant or intermediately susceptible to ceftriaxone were also included in the MDR group [18].

### Plasmid Extraction and Analysis

Plasmid extraction was performed as described by Kado and Liu [19]. Briefly, bacterial cultures from heart infusion broth (HIB) agar plates were suspended in microcentrifuge tubes with lysis buffer, heated for 15 minutes at 70°C and mixed with an equal volume of phenol:chloroform:isoamyl alcohol (25∶ 24∶ 1) for extraction of the plasmid. The supernatants from centrifugation were loaded on a 1% agarose gel in Tris-Acetate-EDTA (TAE) buffer and run two hours at 100 v in 1× TAE buffer. The plasmids were purified by precipitation with two volumes of 100% ethanol and then washed with 70% ethanol twice.

### Plasmid Transformation

Bacterial isolates bearing plasmids were selected to represent isolation sources and bacterial species. The purified plasmid pool from each selected representative isolate was transformed into commercially purchased MAX *E. coli* DH10B (Invitrogen, CA) using the Bio-Rad Gene Pulser Xcell (Bio-Rad Laboratories, Inc, Hercules, CA) with 1 mm cuvettes and pulse conditions of 200 ν and 25 Ω. Transformants were spread onto heart infusion broth (HIB) agar plates containing selected antibiotics. The antibiotic concentrations used were the cut-off point of minimum inhibition concentration (MIC) adopted from the CLSI M-100-S-standard for ampicillin 20 µg/ml, gentamicin 20 µg/ml and tobramycin 20 µg/ml. Three or more single isolated colonies of the transformants for each strain were selected from the antibiotic plates and further streak-isolated for plasmid DNA extraction and characterization.

### Plasmid Size Determination

The representative plasmids were digested with *Eco*RI. Electrophoretic profiles of the *Eco*RI plasmid digests, using *Hin*dIII Lambda DNA as the molecular size marker, were applied to the Fragment Size Calculator (http://www.basic.northwestern.edu/biotools/SizeCalc.html) to estimate plasmid sizes.

### DNA Microarray Detection of AR Genes

Seventeen plasmid samples from 14 individual wild strains (13 MDR and one non-MDR isolate) representing different isolation sources and bacterial species were selected for DNA microarray analysis of antimicrobial resistance genes and of genes for plasmid replication, incompatibility and other plasmid functions. The microarray method for this analysis has been reported previously [10]. There are 1262 gene probes on the array: 775 were designed to detect antimicrobial resistance genes in the NCBI database and 487 gene probes were designed to detect plasmid genes from IncA/C and HI1 MDR plasmids [20], [21]. Microarray probes for the plasmids were designed based on plasmid sequences found in *Yersinia ruckeri* str. YR71 pYR1, *Yersinia pestis* biovar Orientalis str. IP275 pIP1202, *Photobacterium damselae* subsp. *piscicida* pP99-018, *Salmonella enterica* subsp. *enterica* serovar Newport str. SL254 pSN254, *Photobacterium damselae* subsp. *piscicida* pP91278 and *Escherichia coli* p1658/97. Sequences from the pHCM1 MDR plasmid found in *S. enterica* subsp. *enterica* serovar Typhi str. CT18 were also used. The probes were dissolved in 50% DMSO and printed in triplicate onto Corning UltraGAPS slides (Corning Inc., Life Sciences, Acton, MA) using a Qarray mini robot (Genetix, Hampshire, UK) followed by post processing using the manufacturers recommendations (Corning, Inc.) as previously described [20], [21].

Plasmid DNA from the isolates was collected as described above. Random primers and Klenow fragment (New England Biolabs, Beverly, MA) were used to label DNA overnight in a 37°C water bath [10]. Labeled DNA was purified using the Qiagen PCR clean-up kit (Qiagen, Valencia, CA) and hybridized to the microarray over night at 42°C in a Corning hybridization chamber following manufacturer’s recommendations. Microarrays were washed following the manufacturer’s protocol for hybridization with formamide buffer (Corning, Inc.). Array scanning was done using ScanArray Lite, and images were analyzed with ScanArray Express software version 1.1 (Packard BioChip Technologies, Billerica, MA). Hybridizations to the triplicate arrays on each chip were analyzed and scored as previously described. A summary of hybridization data is shown in result, with full data available in Table S1.

### Plasmid Replicon Typing

The purified antibiotic resistance plasmid DNA samples from the *E. coli* DH10B transformants were subjected to replicon incompatibility (Inc) typing by using eighteen pairs of primers to perform five multiplex and three single PCRs which recognized F, FIA, FIB, FIC, HI1, HI2, I1-Iγ, L/M, N, P, W, T, A/C, K, B/O, X, Y and FII replicons as described previously [12].

### Statistics Test

Fisher’s Exact Test was used to test significant trends in the association of plasmid counts and antimicrobial resistance in MDR versus non-MDR bacterial strains by using SAS system software.

## Results

### Antimicrobial Susceptibility and Epidemiological Features of the MDR and Non-MDR *Enterobacteiacea* Isolates

Figure 1 provides the antibiograms and basic epidemiological characteristics of the MDR (A) and non-MDR (B) bacterial isolates tested. Every trauma patient and every patient with an open wound was sampled, as were all patients admitted to the hospital and the operating room (158 patients in total). MDR bacteria were isolated from eighteen of these patients; 16 of these were Iraqi. Four MDR bacterial isolates (#25, #26, #60 and #103) were isolated prior to the new hospital opening, including three from a local Iraqi patient and one from a patient from the Republic of Georgia. It is notable that no MDR bacteria were isolated from the environmental surfaces before patients and healthcare personnel entered the new hospital on 7 December 2007 [16]. However, MDR *Enterobacteriaceae* bacteria were recovered from environmental surfaces soon after the hospital started to accept patients (Figure 1A).

Figure 1A also shows that 100% of the *K. pneumoniae*, *K. oxytoca* and *E. coli*; 66% of the *Enterobacter cloacae* were resistant to 3rd and 4th generation cephalosporins, while 0–12%, 0–31%, and 25–50% of these organisms were resistant to amikacin, gentamicin and tobramycin, respectively. Zero to 47% percent and 6–100% of these organisms were resistant to levofloxin and ciprofloxacin respectively. All MDR bacteria isolates were susceptible to group 1 and 2 carbapenems. A majority (∼85%) of the MDR isolates were resistant to members of the folate pathway inhibitors and trimethoprim/sulfamethoxazole (TRM/SULF).

All non-MDR bacteria isolates, mostly from US and coalition personnel (83%) were susceptible to every antibiotic tested except for ampicillin (81%; this resistance is mostly due to *K. pneumoniae*, which are intrinsically resistant to ampicillin [22]) and TRM/SULF (5% resistant) (Figure 1B). One hundred percent of the *E coli* and *K. pneumoniae* were susceptible to 3rd and 4th generation cephalosporins, levolfoxacin and ciprofloxacin, amikacin, gentamicin and tobramyicn, and group 1 and 2 carbapenems.

### Plasmid Profile and Correlation between the Number of Plasmid and Drug Resistance

**Figure 2:**
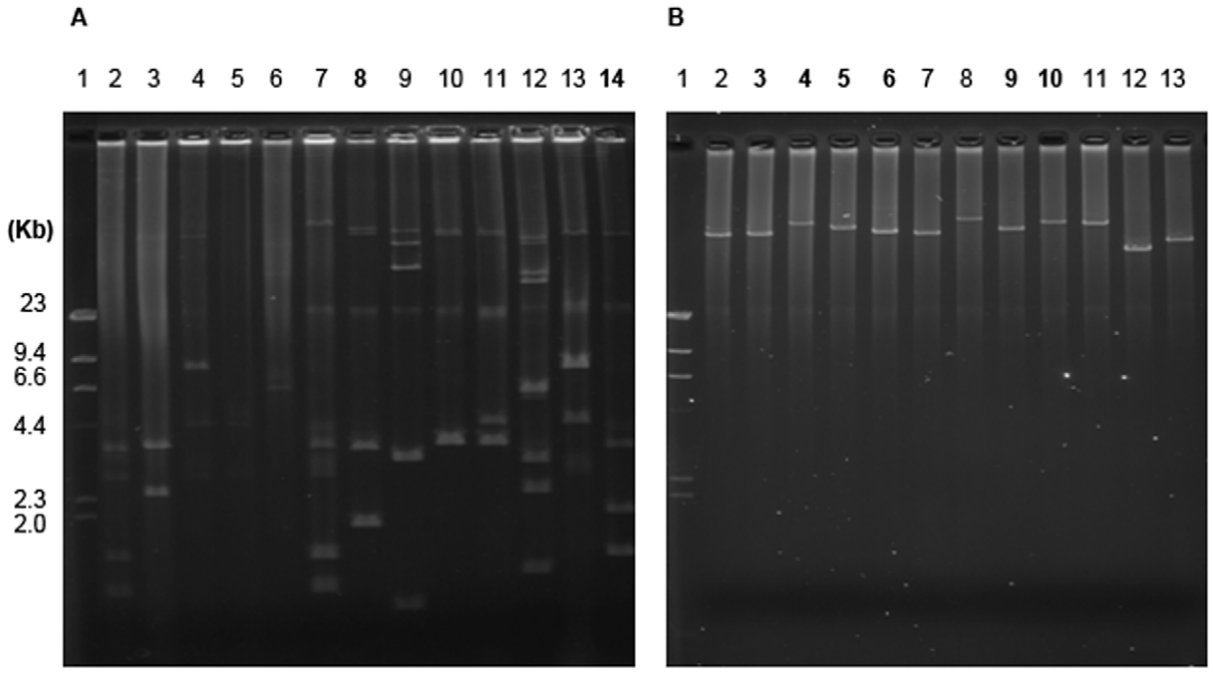
Plasmid profiles of representative MDR isolates and plasmid from transformants. **A**) Plasmids from representative wild MDR isolates. Lane 2: #26*; Lane 3: #144**;** Lane 4:#145; Lane 5: #199; Lane 6: #201; Lane 7: #25; Lane **8**: #**432**; Lane 9:#543; Lane 10:#547; Lane 11:#548; Lane 12:#782; Lane 13:#155 and Lane **14**: #**257**. **B**) Plasmids from transformants of wild isolate plasmid extractions. Lane 2: #A**−150 (∼70 kb); Lane 3: #**A-257**(∼72 kb); Lane 4: #**G-257**(∼98 kb); Lane 5: #**A-432** (∼98 kb); Lane 6: **#G-432** (∼87 kb) ; Lane 7: #A-546 (∼88 kb); Lane 8: #A-351(∼100 kb); Lane 9: #**A-638** (∼77 kb); Lane 10: #**G-638** (∼78 kb); Lane 11: #A-120 (∼72 kb); Lane 12: #A-289 (∼ 52 kb) and Lane 13: #A-575 (∼49 kb) (Plasmids sizes were estimated by the *Eco*RI restriction enzyme digestion, Material and Methods: Plasmid Size Determination.) Both pane1s include the lambda *Hin*dIII DNA size marker in Lane #1. Numbers in bold represent two plasmids carrying multiple drug resistant genes in a single strain;*: strains number of wild parent strains from which -drug resistance plasmids are (their extracted plasmid transformants share the same strain #); **“A”: ampicillin resistant transformant; ***“G”: gentamicin resistant transformant.

Plasmid profiles from all MDR (n = 38) and non-MDR (n = 41) isolates were compared however only the plasmid profiles from representative MDR isolates are presented in Figure 2A to show typical plasmid content. The numbers of plasmids were also determined for each strain (38 strains in total for MDR and 41 for non-MDR bacteria isolates). Table 1 illustrates the number of plasmids detected in the MDR and non-MDR *E. coli* and *K. pneumoniae* bacterial isolates studied. Both the MDR *E. coli* and *K. pneumoniae* isolates harbored significantly more (≥3) plasmids compared to their non-MDR counterparts (≤2). The isolates with more plasmids were significantly more likely to be drug resistant (P<0.0164 for *E. coli* and P<0.0026 for *K. pneumoniae* by the Fisher’s Exact Test, in Table 1). The other *Enterobacteriaceae* isolates were not statistically tested due to their small sample number.

**Table 1:** Number of plasmids in MDR isolates compared with non-MDR isolates.

Number of plasmids present	*E. coli* isolates*		*K. pneumoniae* isolates**	
	MDR	Non-MDR	MDR	Non-MDR
> = 5	7	1	3	1
4	4	0	2	1
3	4	3	7	3
2	1	1	3	7
1	0	5	1	9
0	1	2	0	8
Total isolates	17	12	16	29

* *E. coli*, P< = 0.0164.

** *K. pneumoniae*, P< = 0.0026.

### Characterization of the Plasmids from Multidrug-Resistant Isolates

To investigate the plasmids carrying clinically relevant antimicrobial resistance, extracted plasmids from 14 representative isolates (13 MDR isolates and one non-MDR isolate) were transformed into competent susceptible *E. coli* cells by electroporation. The drug resistance profiles of the extracted plasmid pool transformants that were selected on ampicillin, gentamicin and tobramycin antibiotic agar plates matched the results of the antibiotic susceptibility testing done on these transformants (data not shown). To determine if the antimicrobial resistance genes were located on the same plasmids, we streaked each transformant obtained from ampicillin plates onto gentamicin and tobramycin plates and also streaked each transformant from gentamicin or tobramycin plates onto ampicillin plates. In most cases (∼80%), the antibiograms of the transformants (selected on the three antibiotics) from one antibiotic plate were same as for those selected by the other two antibiotics tested. This was confirmed by antibiotic susceptibility tests on the Phoenix NMIC/ID133 panel, in which most transformants shared similar MICs with their corresponding wild parent isolates (data not shown). However, plasmid transformants of three isolates (#257, 432 and 638) obtained from ampicillin plates did not grow on gentamicin plates, while the transformants from gentamicin plates were also resistant to ampicillin. Gel electrophoresis analysis confirmed that two separate plasmids carrying antimicrobial resistance genes were harbored in the same parent isolates. Figure 2B demonstrates the presence of plasmids of different sizes in transformants selected on ampicillin plates (A-257, A-432 and A-638 in lane 3, 5 and 9) and in counterparts selected on gentamicin plates (G-257, G-432 and G-638 in lane 4, 6 and 10) originated from their three respective parent strains. The sizes of all representative plasmids in Figure 2B were determined by the *Eco*RI enzyme digestion, gel electrophoresis separation of DNA fragments and fragment size calculation (Material and Methods). The smallest plasmid, ∼49 kb, was from the non-MDR isolate A-575, while the size of the remaining 11 plasmids from MDR bacteria isolates ranged from 52 kb to 100 kb (Figure 2B).

### Identification of Antimicribial Resistance Genes by DNA Microarray

Table 2 presents a summary of antimicrobial resistant genes detected by microarray analysis in the 17 plasmids purified from the 14 representative parent isolate strains. All 16 plasmids extracted from 13 of these MDR bacterial isolates carried one to four genes related to aminoglycoside resistance, while a total of five different aminoglycoside resistance genes were identified in these plasmids, including *acc, aadA, aph strA/B* and *ksgA* (Table 2). The plasmids from tranformats resistant to ampicillin alone (A-257, A-432 and A-638) lacked the *aac* gene, while the transformants resistant to gentamicin alone (G-257, G-432 and G-638) and the transformants resistant to both ampicillin and gentamicin carried the *aac* gene known to confer gentamicin resistance [23].

**Table 2:** Summary of isolate hybridizations to microarray gene probes^a^.

ID^b^	Strain origin	Aminoglycosides	β-lactams	Chloram-phenicol	Sulfameth-oxazole	Tetracycline	Trimetho-prim	Transfer associated	Replicon types
**A103**	*K. pneumoniae*	*acc, aadA, aph, strA/B*	*tem*1	*cat*	ND	ND	*dfrA*	*insA,B,C3,D1,E1,* IS1, *tnpA, tnpR*	*–*
**A150**	*K. pneumoniae*	*aac, aadA, strA/B*	*oxa-*1, *tem*1	ND	ND	ND	*dhfR*	*insA,B,E, tnpA, tnpR*	*–*
**A257**	*E. coli*	*aadA*	*tem*1	ND	ND	ND	*dfrA*	*insA,B,C3,E1, tnpA, tnpR*	F, FII
**G257**	*E. coli*	*aac,, aadA, strA/B*	*oxa*-1	ND	ND	*tet*(A), *tetR*	*dfrA*	*insA,B,C3,E1, intI1, tnpA*	FII, FIA, FIB, F
**A432**	*E. coli*	*aadA*	*tem*1	ND	*sulI, sulII*	ND	*dfrA*	*insA,B,E1, intI1, tnpA, tnpR*	B/O, K
**G432**	*E. coli*	*aac*	*ampC, tem*1, *ctx-m-*15,	ND	ND	ND	ND	*insA,B,E1, tnpA,*	I1
**A546**	*K. pneumoniae*	*aac, ksgA*	*ctx-m-*15, *tem*1	ND	ND	ND	ND	*insA,B,E1, tnpA*	I1
**A25**	*E. coli*	*aac, aadA*	*oxa-1, vim-2*	*cat*	ND	*tet*(A), *tet*(B), *tetR*	*dfrA*	*insA,B,E1, intI1, tnpA*	FII, FIA, FIB, F
**A151**	*K. pneumoniae*	*aac, aadA, aadB, ksgA*	*ampC, oxa-1, shv, tem*1	ND	ND	ND	ND	*insA,B,D1,E1, tnpA, tnpR*	*–*
**A202**	*K. pneumoniae*	*aadA, strA/B*	*ctx-m*-15	ND	*sulII*	ND	*dfrA, dhfR*	*insA,B,D1,E1, intI1, tnpA*	N
**A199**	*E. coli*	*aac, aadA*	ND	ND	ND	*tet*(A), *tet*(B), *tet*(C), *tetR*	*dfrA*	*insA,B,C3,D1,E1, intI1, tnpA,*	FII, FIB,
									F
**A351**	*E. cloacae*	*aac, aadA, aph, strA/B*	*ampC, oxa-*1, *ctx-m-*15, *tem*1	ND	*sulII*	ND	*dfrA, dhfR*	*insA,B,C3,D1,E1, intI1, tnpA, tnpR*	*–*
**A521**	*K. pneumoniae*	*aac,, aadA, aadB, aph, strA/B*	*ampC, tem*1, *ctx-m-15*	*cat*	*sulII*	ND	*dfrA*	*insA,B,B2,C3,D1,E1, intI1, tnpA, tnpR*	*–*
**A638**	*K. pneumoniae*	*ksgA, strA/B*	*ampC, shv*	ND	*sulII*	ND	*dfrA*	*insA,B,D1,E1, tnpA*	*–*
**G638**	*K. pneumoniae*	*aac, aadA, aphA, strA/B*	*tem*1	*cat*	ND	ND	*dfrA*	*insA,B,C3,D1,E1, intI1, tnpA, tnpR*	*–*
**A289**	*K. oxytoca*	*strA/B*	*oxa-*1	ND	*sulI*	ND	*dhfR*	*insA,B,C3,D1, intI1, tnpA*	N
**A-575**	*E. coli*	ND	*tem*1	ND	ND	ND	ND	*insA,B,E1, tnpA, tnpR*	F, FII

a Genes and gene families with multiple probes are summarized and presented only once;

b “A” in front of ID indicates the transformants were obtained from ampicillicn plates, “G” means transformants were obtained from gentamicin plates; ND indicates that none of the gene probes in this table hybridized to DNA from the isolate (full hybridization data is available in supplemental table S1).

One to four genes conferring resistance to β-lactams were detected in all transformants except for A-199. A total of six β-lactamase genes (*bla*_TEM1_, *bla*_AMPC_, *bla*_CTX-M-15_, *bla*_OXA-1_, *bla*_VIM-2_ and *bla*_SHV_) were identified on plasmids in all the isolates in the study. The *bla*_TEM1_ gene was detected most frequently (11 out of 16 plasmids) followed by the *bla*_CTX-M-15_ (5/16), *bla*_OXA-1_ (5/16) and *bla*_AMPC_ (5/16) genes. The *bla*_SHV_ gene was only detected in A151 and A638, which originated in *K. pneumoniae* isolates. Most of the plasmids studied (13/16 plasmid transformants) carried the trimethoprim resistance genes: *dfr*A/R and sulfamethoxazole genes *sul*I and *sul*II were detected in six of the plasmids tested. The chloramphenicol resistance gene (*cat*) and tetracycline resistant genes (*tet*) were only detected in four and three of plasmids tested, respectively (Table 2).

A plasmid from a non-MDR isolate (*E.coli* #575) was found to carry only the *bla*_TEM1_. However, plasmids from both MDR (n = 16) and non-MDR (n = 1) isolates carried four to eight transfer associated genes (Table 2). Nine out of 16 plasmids (56%) from MDR isolates carried the class 1 integrase gene (*int*I1), which is often associated with antibiotic resistance among Gram-negative bacteria [24]. The microarray also assayed for genes found on the “backbone” of two families of MDR plasmids found in Gram-negative bacteria, IncA/C and IncHI1. Out of 487 plasmid genes, only 86 genes scattered along the backbones of these plasmids were detected indicating that IncA/C and IncHI1 plasmids are not found in these isolates (data not shown). Most of the genes detected were associated with antimicrobial resistance or mobile genetic elements and are listed in Table 2 (complete hybridization data is in Table S1).

### Replicon Typing of Plasmids

Among the seventeen plasmids from the fourteen *Enterobacteriaceae* isolates (mostly *E. coli* and *K. pneumoniae*, Table 2), ten had one or more replicon targets detected, while seven plasmids did not have any of the published replicon gene targets (Table 2). All seven plasmids originating from *E. coli* isolates were typeable and were found carrying 1∼4 replicon targets respectively (Table 2). Seventy percent of plasmids (five out of seven) from *E. coli* carried IncF and FII. Six out of eight plasmids from *K. pneumoniae* were not typeable using this method. The remaining two plasmids from *K. pneumoniae* isolates were detected as IncI1 and IncN. The plasmid from one *E. cloacae* strain was also not typeable, while the plasmid from *K. oxytoca* was positive for the IncN as was one of the *K. pneumoniae* isolates (Table 2). Diverse replicon types were identified for the two plasmids harbored in the same parent strain (#432) such as IncB/O and K in plasmid A432 and IncI1in plasmid G432. Although A257 and G257 both shared IncFII, G257 carried two additional F replicon markers, IncFIA and FIB (Table 2).

## Discussion

To our knowledge, this is the first report of the characteristics of plasmids found in MDR *Enterobacteriaceae* compared to their susceptible counterparts isolated from prospective surveillance of patients, healthcare workers and environmental surfaces in a new Iraqi hospital. In this study, we focused on the impact of the plasmids on drug resistance and the genetic features of the plasmids harbored in these multidrug resistant bacteria. Due to the instability and compromised infrastructure, molecular epidemiologic surveillance is difficult in this region of the world. The information provided here furthers our understanding of the mechanisms of drug resistance in this specific region and their evolutionary relationship with other parts of world.

DNA microarray analysis revealed various ESBL resistance genes in these *Enterobacteriaceae* strains isolated in Iraq. The globally disseminated *ctx-m*-15 gene, which is associated with the current epidemiology of ESBLs [5], and two other ESBLs related genes, *bla*_OXA-1_ and *amp*C, were detected in approximately one third of the plasmids we tested (Table 2). The *tem-*1 gene, a commonly-encountered β-lactamase gene in Gram-negative bacteria, which confers up to 90% of the ampicillin resistance found in *E. coli* [25], was found in both MDR and non-MDR isolates. The *vim*-2 carbapanemase gene was detected from A-25 although the phenotype of both transformant (#A-25) and parent strain (#25) were imipenem susceptible. It may be explained by mutation of the gene or lack of its expression in this isolate. A plasmid sequence project is ongoing and might answer this question in the near future. All plasmids from MDR isolates carried genes encoding resistance to ≥3 classes of antibiotics including resistance to aminoglosides, β-lactams, trimethoprim/ sulfamethoxazole, tetracycline and chloramphenicol. This result is also consistent with previous studies that multiple drug resistance genes were carried on a single plasmid [5], [9], [26], which could be important vectors for dissemination of multidrug resistance by the horizontal transfer through natural mechanisms such as conjugation or transformation [4]. Most transformants shared similar MICs with their corresponding parent strains (data not shown), implying that plasmids played a significant role contributing to the multidrug resistance of the phenotypes of these *Enterobacteriaceae* isolates. Although the intergrase gene (*int*I1), the antibiotics resistant maker in Gram-negative bacteria [24] was only detected in MDR isolates, other transfer associated genes, such as insertion sequences and transposons were detected in both MDR and non-MDR isolates (Table 2). Therefore, the transfer related genes located on plasmids can also be a potential factor that impacts dissemination of the drug resistance through mobilization, acquisition and assembly of foreign resistance genes between bacteria existing in hospitals, communities and the environment [27].

In this study, seven Inc replicon types (IncFII, FIA, FIB, B/O, K, I1 and N) were detected among 10 plasmids isolated from eight parent *Enterobacteriaceae* isolates. These seven replicon types have been reported to be mainly carried by plasmids in *E. coli*, *K. pneumoniae* and some *Salmonella enterica* in many regions of the world [5]. However, plasmid replicon typing has not been previously reported on isolates from the Middle East. The homologous replicon types in plasmids from *Enterobacteriaceae* isolates in Iraq such as IncF and FII, found in many parts of the world, suggested a similar evolutionary origin. The plasmid families IncA/C and IncHI1, widespread in North America and Asia respectively, were not found in these isolates implying geographical diversity for MDR plasmids in Iraq [5], [13]. Although the replicons identified in this study did not appear to be related to a specific resistance genotype, the data collected will help in understanding the origin of these plasmids and provide information for the international community studying MDR plasmid evolution and spread. Plasmids from one *E. cloacae* and six of eight *K. pneumoniae* isolates did not have any of the 18 replicon types detected. This may indicate different evolutionary origins of these plasmids, and could be due to the geographic distance between *Enterobacteriaceae* isolates in this study and the previous studies [5]. In addition, the established method used in the current study only covers the 18 most prevalent of the 26 known Inc replicon types in the *Enterobacteriaceae* [5], [13].

Although we cannot clearly identify the origin of these MDR plasmids, the selective pressure provided through high antibiotic use in countries where their use is not controlled (*e.g.* by prescription) including Middle Eastern countries such as Saudi Arabia [4] and Iraq [28] could be an important factor for high community MDR bacterial colonization and community dissemination. Since the majority of the MDR bacteria in our study were isolated from local Iraqi patients, and this facility was their first point of entry into the healthcare system, these events /findings might reflect the baseline or ambient colonization in the local community [16]. In our previous study, we found that MDR *K. pneumoniae* strains 636, 640 (both from Iraqi patients) and 614 (from hospital enviroment); *K. pneumoniae* strain 691 (from an Iraqi patient) and 713 (from hospital envroment); and *E. coli* strain 732 (from a US heath care worker) and 763 (from hospital enviroment) are clonally related based on PFGE analysis [16], which was supported by the identical MDR phenotype (Figure 1A) and similar plasmid profiles (data not shown) determined in this study. These results strongly suggest that the MDR bacteria were introduced into the hospital environment by Iraqi patient carriers since there were no MDR bacteria detected before the hospital opened. Additionally, MDR plasmids could be acquired by susceptible bacteria during treatment with antibiotics that can induce and select for horizontal transfer [29]. Therefore, if the MDR bacteria were brought into hospitals by patient carriers this can be seen as a potential risk factor for initiating nosocomial contamination and spread, as seen in our study (Figure 1A ) and also in other reports [16], [30]. This study also highlighted that multiple plasmids are found in these MDR bacteria, and can be a possible source of rearrangement of antimicrobial resistance genes into new combinations and new genetic elements such as plasmids with different Inc groups. Additionally, the presence of multiple replicon types in a single organism allows greater opportunity for dissemination of plasmids.

In conclusion, this is the first report of the characteristics of the plasmids found in MDR *Enterobacteriaceae* compared to their susceptible counterparts isolated in Iraq. The large plasmids, carrying resistance genes and transfer-associated genes, may be potential factors for regional dissemination of antibiotic resistance.

## Supporting Information

Table S1 Presence and absence of antimicrobial resistance genes and plasmid backbone genes in the 17 selected plasmids from Iraq *Enterobacteriaceae* isolates as determined by DNA Microarray. Blue: absence of genes; Red: presence of genes.
(XLS)
